# Supplementary material for: A feasibility study to test a novel approach to dietary weight loss with a focus on assisting informed decision making in food selection
Source: PLoS One. 2022 May 26;17(5):e0267876. doi: 10.1371/journal.pone.0267876 (PMC9135285; doi:10.1371/journal.pone.0267876)
Supplement: S1 Appendix — (PDF) [file pone.0267876.s003.pdf]

S1 Table. iDip educational session topics

Dietary Improvement Session Topics	
**1**	FFQ, starting weight loss
**2**	Weight monitoring
**3**	24-hour dietary record 1
**4**	Individual advising 1
**5**	Protein 1
**6**	Establishing routine and 24 hour dietary record 2
**7**	Fiber 1
**8**	Individual advising 2
**9**	Physical activity
**10**	Protein 2
**11**	Fiber 2
**12**	Trouble shooting for slowing down
**13**	Peer experience sharing
**14**	Weight maintenance: Difference between weight loss and maintenance
**15**	Weight maintenance: Building a Healthy Plate
**16**	Weight maintenance: Hidden Kcal Contributors and Diet Foods
**17**	Barriers to Healthy Eating
**18**	Fats
**19**	Individual advising 3
**20**	Salt and Potassium
**21**	Eat the Rainbow: The Importance of Vitamins and Minerals
**22**	Final review
