# Supplementary material for: A feasibility study to test a novel approach to dietary weight loss with a focus on assisting informed decision making in food selection
Source: PLoS One. 2022 May 26;17(5):e0267876. doi: 10.1371/journal.pone.0267876 (PMC9135285; doi:10.1371/journal.pone.0267876)
Supplement: Attachment — Submitted filename: Response to Reviewers.docx [file pone.0267876.s007.docx]

**Response to Reviewers**

Thank you for your careful evaluations of our manuscript and helpful comments. As shown below, we addressed all of the comments and revised the manuscript accordingly. With this revision, we believe that descriptions of the study are clearer, and the manuscript is easier to follow.

**Additional Editor Comments:**

**The title of the manuscript inaccurately describes the study. The paper needs the be re-written for syntax.**

- Title has been revised to better summarize the study.

**ABSTRACT
The abstract methodology is vague. What makes this study individualized as described in the title? What was the breakdown of male and female participants and average age? The results are descriptive, with no mention of p-values. Strong conclusions for n=12 finishing the iDip program.**

- Clarification of individualization: In lines 5-8, the abstract has been reworded to emphasize the self-selected dietary changes by individual participants.
- Average age and gender proportion of the participants are included in line 15.
- P-values are added for results.
- The conclusion relates only to the feasibility of the approach and does not make claims beyond that.

**INTRODUCTION
Lines 44-45: These are outdated programs that do not have as large of following as the did when first launched and the authors should include updated diet programs as examples.**

- Excluding certain food groups are a mainstay of dieting books although popularity may come and go. Atkins and Zone diets are still used by many. More importantly the new and trendy diets are often rebranding of old diet programs. For example, the keto diet, which is currently very popular, is essentially the same as the Atkins diet although it is marketed as novel. The paleo and plant-based diets are similar to Zone and Ornish diets, respectively. This point is added to the Introduction in lines 43-48.

**Several weight loss/energy reduction methods are described in the introduction. However, the title describes iDip as an individualized program and the introduction should address the importance of addressing individualized needs for successful weight loss.**

- The last paragraph (line 80-94) of the Introduction is revised to highlight the individualized approach of our study.

**MATERIALS AND METHODS
Lines 88-132 should come after participant recruitment and study design. This section is actually part of study/intervention design.**

- This section has moved to line 146-191.

**CONSORT guidelines should be followed**

- After confirming with the PLOS ONE editorial office, the TREND checklist should be used due to the non-randomized study design.

**Were medications, alcohol intake, or tobacco use taken into consideration for recruitment?**

- Medications (except for insulin), alcohol intake, or tobacco use were not taken into consideration for recruitment as described in Participant recruitment.

**No clear power analysis is presented.**

- Details of power calculation to determine the sample size were added to lines 106-113.

**Line 150- reference needed for attrition.**

- Reference is added in line 112.

**It is unclear how this study was individualized apart from the 3, 30-minute session offered over the 12 month intervention.**

- What we meant by “individualized” was that individuals decide and create their weight loss diet, not that instructors provide several one-on-one advising sessions. This point is clarified in both the Abstract (Line 5-8) and Introduction (Line 82-90).

**Line 164: be specific beyond periodic. If the objective was to direct food intake based on protein and fiber density, then this feedback seems like it should be more regular than periodic.**

- Timepoints were added in line 141.

**Line 183: again, be specific about periodic.**

- Timepoints were added in lines 208-209.

**How often were participants providing feedback on home weighing?**

- The weight chart was provided to participants on a weekly basis as mentioned in line 140.

**The fiber and protein density plots seem difficult for the lay-person to understand. How was comprehension amongst participants assessed?**

- Based on “Improvements in recall and food choices using a graphical method to deliver information of select nutrients,” reported by Pratt et al., the comprehension of the plot was high as 83.8% of participants (n=142) responded that they understood the nutrition graph.
- Our participants also reported the visual feedbacks were easy to understand and helpful as mentioned in lines 331-332.

**How were FFQ collected? Digitally?**

- The FFQ was collected in a paper version and we clarified this point in lines 205-206.

**RESULTS
Exit survey could become supplemental material**

- Exit survey was moved to the supplemental materials, as S3 Appendix.

**DISCUSSION
It is unclear how iDip is innovative.**

- We have revised the Abstract (lines 5-8), Introduction (43-48, 56-59, 80-94), Methods (146-191) to clearly state our innovative approach in the early part of the manuscript.

**Journal Requirements:**

**When submitting your revision, we need you to address these additional requirements.**

**1. Please ensure that your manuscript meets PLOS ONE's style requirements, including those for file naming. The PLOS ONE style templates can be found at**

<https://journals.plos.org/plosone/s/file?id=wjVg/PLOSOne_formatting_sample_main_body.pdf> and

<https://journals.plos.org/plosone/s/file?id=ba62/PLOSOne_formatting_sample_title_authors_affiliations.pdf>

**2. In your Methods section, please provide additional information regarding some discrepancies found between your submission and the clinical trial protocol. For example, we note that the sample size in the protocol is reported to be 180; in the CT.gov registry, it is mentioned that 44 participants were recruited; while in the manuscript text, the sample size is 15. Please explain these discrepancies, and provide more detail on the how the sample size was calculated. Furthermore, we note that, according to the protocol, the dietary intake assessment should have been carried out at 0, 6 and 12 months; while in the manuscript, it is reported that it was carried out only at baseline and at 12 months. Also, please clearly state the dates for participant recruitment and follow-up.**

- IRB approved the iDip as a part of a series of studies.
- The registry has been revised to publish each trial separately. The first study described in this manuscript is now registered with 14 participants.
- Dates are specified in lines 96-102.

**3. Please include additional information regarding the survey or questionnaire used in the study and ensure that you have provided sufficient details that others could replicate the analyses. For instance, if you developed a questionnaire as part of this study and it is not under a copyright more restrictive than CC-BY, please include a copy, in both the original language and English, as Supporting Information. Moreover, please include more details on how the questionnaire was pre-tested, and whether it was validated.**

- We used a validated FFQ (S2_Appendix) as shown in lines 205-208.
- We appreciate your kind suggestion. Exit survey is now S3_Appendix.

**4. Please note that according to our submission guidelines (**[**http://journals.plos.org/plosone/s/submission-guidelines**](http://journals.plos.org/plosone/s/submission-guidelines)**), outmoded terms and potentially stigmatizing labels should be changed to more current, acceptable terminology. For example: “Caucasian” should be changed to “white” or “of [Western] European descent” (as appropriate).**

- In Table 1, we have revised to White from Caucasian.

5. **Thank you for submitting your clinical trial to PLOS ONE and for providing the name of the registry and the registration number. The information in the registry entry suggests that your trial was registered after patient recruitment began. PLOS ONE strongly encourages authors to register all trials before recruiting the first participant in a study.**

**As per the journal’s editorial policy, please include in the Methods section of your paper:
1) your reasons for your delay in registering this study (after enrolment of participants started);**

- We mentioned our reason for our delay in lines 98-99.

**2) confirmation that all related trials are registered by stating: “The authors confirm that all ongoing and related trials for this drug/intervention are registered”.**

- We made a confirmation in lines 99-100.

**Please also ensure you report the date at which the ethics committee approved the study as well as the complete date range for patient recruitment and follow-up in the Methods section of your manuscript.**

- The report date at which the ethics committee approved and the complete data range for recruitment and follow-up are stated in lines 97, 100-101.

**6. We note that you have stated that you will provide repository information for your data at acceptance. Should your manuscript be accepted for publication, we will hold it until you provide the relevant accession numbers or DOIs necessary to access your data. If you wish to make changes to your Data Availability statement, please describe these changes in your cover letter and we will update your Data Availability statement to reflect the information you provide.**

- DOI is 10.13012/B2IDB-4710255_V1.

**Reviewer #1**:

**The title is too long and should reflect the fact that it is a pilot study to test the feasibility of iDip on weight loss at 12 months.**

- Removed Individualized Dietary Improvement Program (iDip) in title and added “Feasibility”.

**The initial protocol stated that, 30 participants would be in an intervention group and 30 in the wait-list group. The study design reported in this paper involved only 12 participants in a before-after comparison. Please explain.**

- Because of the feasibility nature of the study and logistic limitations, we started with half the size of original plan, n=14 instead of n~30, and planned to repeat the procedure if necessary. However, we were able to achieve the primary objective with the first cohort. Thus, rather than repeating the same trial, we amended the IRB protocol for the second trial, which is currently ongoing. Also, because the number of wait-listed participants declined in the process, we had to adopt the before and after design instead.

**Rather than using the minimum sample size reported in other published studies, a formal power calculation should be provided to support the selected sample size. With more than one primary outcomes, what was the minimum effect size to detect post intervention?**

- Details of the power calculation to determine the sample size were added to lines 106-113.

**Data analysis only considered the paired t-tests on the differences before and after the intervention, and Pearson correlation on the associations between body weight and protein/fiber density. For a small sample of 12 participants who completed the intervention, have the authors checked the distribution of outcomes and considered non-parametric tests? The authors also compared the two groups of participants between the top and bottom half in weight loss at 12 months, what test was performed here? The repeated measures ANOVA reported in Figure 7 should also be mentioned in statistical analysis section.**

- Lines 226-228 are added to show enhanced detail on statistical analyses including distribution of outcomes.
- We did not consider non-parametric tests because Shapiro-Wilk testing indicated our data set was normal.
- Top and bottom groups were divided into two halves by the magnitude of weight loss. No statistics should be applied between their weights. We did run statistics on diet between the two groups (Fig 7). As mentioned in the captions, we used paired t-test.
- The repeated measures ANOVA is added in the methods section.

**As this is not a randomised trial, baseline demographics and medical histories can be reported in Table 1 on the 12 participants who completed the intervention at 12 months. The two participants that discontinued the intervention can be described separately in text. It is also important to present baseline characteristics of the two groups of participants above or below 5% weight loss at 12 months and see how they may differ.**

- We added another column to show the characteristics of completers in Table 1.
- No significance was found in any demographic data between the top and bottom groups in weight loss. A sentence is added to Results in lines 242-243.

**The raw outcomes presented in Table 2 would be better presented as a full dataset at baseline and 12 months, since the primary and secondary outcomes were measured at these two time points on a total of 12 participants.**

- We believe adding 6 and 18 month data is informative.

**There was no Table 3. For Table 4, I would suggest reporting median together with mean (SD) for a score of 1-7. The SE is redundant and should be removed.**

- We apologize for our mistake and appreciate your pointing this out. We have changed Table 4 to Table 3 and eliminated SE. Table 3 has become S3 Appendix.

**Both Pearson and Spearman correlation coefficients should be reported in a small sample.**

- Because Shapiro-Wilk testing indicated our sample, while small, was normal. As such, we do not find it necessary to utilize non-parametric testing.

**Note that mean weight loss is different from % weight loss, and the terms should not be used interchangeably.**

- Revisions have been made throughout the manuscript to not to use these terms interchangeably.

**Reviewer #2:**

**This paper reports on a small study evaluating the Individualized Diet Improvement Program (iDip) at 12 months. The 14 participants are only evaluated pre and post.

Major
1. Study design
This study lacks a comparison group which makes the authors' claims about the success of the program and the critical role of certain aspects of the program (the plot) unfounded. This evaluation is also only completed on a small group of people. If the sample were small, but the study design more rigorous, this alone would not be a major issue. However, in combination, it severely impairs any contribution to scientific knowledge.**

- This is a feasibility study to test if the novel, dietary approach can deliver a mean weight loss of >5% initial body weight. A before-and-after design without a control group is frequently used for weight loss studies of 12 months or less because it is very unlikely that a control group achieves >5% weight loss without any intervention in this time span.
- Efficacy of the PF plot for a food selection by customers in a restaurant setting was demonstrated in our previous study “Improvements in recall and food choices using a graphical method to deliver information of select nutrients,” reported by Pratt et al. Applying the PF plot in achieving weight loss was part of the study objectives, but we did not find any overstatement of our study results regarding the PF plot’s efficacy in our manuscript. Based on the editor and the other reviewer’s comments, we revised to present the innovative nature of our approach including the PF plot more clearly in Abstract (lines 5-8) and Introduction (lines 82-90).
- Details of the power calculation to determine the sample size were added to lines 106-113.

**2. Intervention
It is unclear what exactly is novel and innovative about this weight management approach. The Protein-Fiber plot seems to be a big part of it. There is no evaluation of whether this plot was really used and all participants received it, so it is impossible to know its actual contribution to the program. The plot itself also appears to present only single foods and does not seem to account for whole diet or mixed foods (like casseroles) which is a major limitation.**

- As mentioned in the reply to the previous query, the innovative approach is better highlighted throughout the revised manuscript
- PF plot is the only analysis and feedback on participants’ diet. Participants were able to create and advance their weight loss diet based on the plot. No single recipe, food exclusions, or meal plans were provided to them. This point is clarified in the Methods section in lines 142-143 and 350-351.
- As shown in Figures one, five and six as well as Pratt et al 2016, the advantage of the PF plot is it can plot individual foods, a dish (like casserole), meal, or meals. Alternatively, a dish can be plotted by individual ingredients as well to improve a recipe. In a previous study (Pratt et al), there were significant improvements in cafeteria food choices when the plot was displayed, whereas the presence of a nutrition facts label had no effect. Additionally, the blue total dot, as mentioned in Figure one caption, displays the meal or day’s average nutritional value.

**In the discussion, the study is described as innovative because it does not use calorie counting, specific food advice and is non-restrictive. But, ultimately the program applies a 25% energy restriction.**

- The expression “non-restrictive” may be misleading. We replaced it with “no exclusion of certain food groups.” This point is clarified by rewording in lines 405 and 414.
- We made it clear to the participants that their daily energy intake must be reduced to achieve weight loss. This is the rationale for the two target boxes for weight maintenance and weight loss in the PF plot (Figure one and lines 163-168). However, the participants reduced the energy intake by choosing from green foods in the PF plot to move their meal closer to the target weight loss box. Energy balance was monitored via daily self-weighing. The difference is that participants achieved negative energy balance without traditional daily calorie counting or following portion controlled daily menus.

**In the discussion it says that participants were "encouraged to include all foods and self-select to moderate intake of foods with low (high) protein and fiber densities".**

- We rephrased it to make our point clear as follows: “Participants were encouraged to self-select to limit intake of foods that contain minimal fiber and protein, but they were not instructed to exclude any particular foods from their diet.” (lines 358-360)

**Given that the program included 19, 60-min meetings and 3 individual sessions delivered by a registered dietician, it would appear that this program is little more than a group-based, dietitian program.**

- We assume the reviewer is questioning the individualized nature of our program. What we meant by “individualized” was that individuals decide and create their weight loss diet, not that instructors provide several one-on-one advising sessions. This point is clarified in Abstract (lines 5-8) and Introduction (lines 82-90).

**Despite suggestion that this program lacked detailed food or intake monitoring, the participants did monitor their food intake by completing 6 different 24h recalls. Although not a formal part of the program, with no comparator, it is impossible to know the effects of this. Previous studies have shown the importance of monitoring and how it alone can reduce intake.**

- The participants achieved significant weight loss with only six 24-hour records in the 12-month period and daily weighing (this is a daily monitoring although not food intake monitoring). An important difference is time required for participants for 6 24-hour records and daily weighing was much less than daily food intake monitoring. Previous studies did show monitoring alone could reduce intake. However, to our knowledge, there is no study that reported weight loss success comparable to ours in magnitude, duration, and retention by daily food intake monitoring alone.

**Minor
1. Why does the program include daily weighing? This can be a controversial recommendation that can result in obsessing over insignificant changes.**

- As stated in lines 178-184, daily weighing is the easiest and fastest way to monitor energy balance. “Frequent self-weighing with electronic graphic feedback to prevent age-related weight gain in young adults,” by Bertz et al. has shown that providing weekly feedback prevents weight gain in young adults. Daily weighing shows the fluctuations but more importantly displays an overall trend. We emphasized that participants should look at weekly and monthly trends, not daily fluctuations. Our participants reported no problems with daily weighing.

**2. Figure 8 is an R output that may not be easily interpretable by many readers.**

- The matrix was used in Figure eight as it displays relatively minor findings and saves space.

**3. In the method, it states that dietary recalls were "periodically" completed. This needs to be specified.**

- The months were data was collected are specified in the legend of Figure seven, and also added to the methods (lines 141 and 208-209).

**4. The intervention and its specific novelty is not made particularly clear. Parts of the discussion provide better insight than the method, but this should be clear long before**

- Thank you for your helpful comment. We have revised the Abstract (lines 5-8), Introduction (43-48, 56-59, 80-94), Methods (146-191) to state our innovative approach clearly in the early part of the manuscript.

**5. The expenditure side of energy balance is not acknowledged in the introduction.**

- If the “expenditure side of energy balance” refers to exercise, it is very challenging to achieve the large negative energy balance necessary for weight loss via physical activity only. As such, we focused on the dietary aspect of weight loss in our program.

**6. The discussion describes the diet as non-restrictive. It includes a 25% restriction.**

- What we meant by “non-restrictive” was not to exclude any food groups from diet. This point is clarified by changing wording in lines 405 and 414.

**7. There is no citation for the Power calculation.**

- The details of power calculation to determine the sample size were added to lines 106-113.

**8. There may exist an opportunity to explore how participants chose to restrict their diet and whether certain patterns were more successful. This could be more interesting as an outcome of the study and more of a contribution to the field.**

- Thank you for an interesting suggestion. We will explore it in future, larger studies.
