# Supplementary material for: A feasibility study to test a novel approach to dietary weight loss with a focus on assisting informed decision making in food selection
Source: PLoS One. 2022 May 26;17(5):e0267876. doi: 10.1371/journal.pone.0267876 (PMC9135285; doi:10.1371/journal.pone.0267876)
Supplement: Attachment — Submitted filename: Response to Reviewers.docx [file pone.0267876.s008.docx]

**Response to Reviewers**

We truly appreciate your thoughtful review of our manuscript and helpful comments. As shown below, we addressed the comments and revised the manuscript accordingly. With this revision, we believe that descriptions of the study are clearer, and the manuscript is easier to follow.

**Additional Editor Comments:

As a reviewer notes, the tone of the manuscript needs to ensure that it is a feasibility study without a control group. The lack of a control group is a study limitation that needs to be stated. Please ensure the reviewer responses are addressed. Use of terms such as non-randomized and single arm trial would be beneficial throughout the manuscript including the conclusions.**

- Thank you for your kind suggestion. We stated non-randomized and single-arm trial in lines 123, 343, and 433.
- We do not consider the lack of a non-treatment control group as a major limitation of this study. Although we agree that a randomized and controlled trial (RCT) is a gold standard of clinical studies, in the case of weight loss study, a large-scale RCT (DPP Research Group, 2002) has shown that the weight of the placebo group was unchanged after one year, whereas metformin and lifestyle group significantly lost weight. This point was added to the Discussion in line 416-421.

**Further, the pilot or feasibility consort should be utilized. Also, the (TIDieR) checklist and guide should be used to describe the intervention.**

CONSORT for pilot and feasibility study

- We added the timeline of intervention in line 131-132 to follow the guideline.
- We added the CONSORT as S4 Appendix.

TIDieR

- We added the objective of the study in line 125-127 and the location of study administration in line 131.
- We added the checklist as S5 Appendix.

**Reviewer #2:**

**Major**
**Issues raised about the study design previously remain. Without a control group, the authors cannot conclude that the PF plot has efficacy over and above the methodological aspects of the study which included an extensive amount of dietary recall and FFQ and dietary coaching with a health professional. Further, as a feasibility study, it lacks any in-depth evaluation of the innovative components of the intervention (eg, the PF plots).**

- Thank you for raising questions, which seem to be due to unclear descriptions of our objectives and intervention protocol.
- The objective of this study is not to compare the efficacy of our approach with certain conventional approaches. The objective of this feasibility study is to determine if our new approach that replaced conventional methods works. Revisions are made to clarify this point in the revised last paragraph (line 80-87) of Introduction, Study Deign (line 125-127; 129-132; 141-144), and Development of novel learning tools (line 147-151; 169-172) in Methods
- For this objective, a control group should be a non-treatment group, not a certain traditional weight-loss method. The lack of non-treatment control is addressed in the reply to the editor above.
- Although graduate students with RDN credentials in this research project provided lectures and coaching, the entire interactions in both group sessions and individual advice were performed to test the new approach:
  - Feedback of FFQ, 24-hour records were always provided using the PF plot.
  - No food was excluded from participant’s diet, and even no recipe was provided to them. As the manuscript title shows, what dietitians provided was a visual comparison of food properties for participants to make informed decisions.
  - Efficacy of visualization of nutrient values with PF plot was previously reported (Pratt, 2016).
- These points were stated clearly in the revised Methods (lines 125-127, 137-144, and 147-151) and Discussion (lines 352-366 and 368-381).
- In-depth evaluation of innovative components would be optional because weight loss was set as the primary outcome in our study design. This reviewer’s comment overlaps with the comment on the exit survey, which is addressed below.

**The authors state that they could not find any overstatement in their conclusions. Two concrete examples are provided below. Each shows a lack of acknowledgement of study limitations while also being worded too strongly.
1) “The individualized PF plot played a critical role in dietary improvement and allowed participants to use the knowledge gained from education sessions to self select foods to increase or decrease.” (line 404-406)**

- The wording of the statement is revised to reflect our results accurately (line 367-368).

**Regular and intensive dietary recall and meetings with a dietician were much more likely the cause of this and the study design does not allow these to be teased apart. There is no evaluation of the possible mechanisms either. This statement suggests a linear process that has been demonstrated. The study provides no data to support this strongly worded statement. Furthermore, the ethics application notes that the purpose of the dietary recall is to “check adequacy of their diet and to troubleshoot if a participant is not losing weight effectively” so the recall process is seemingly a significant part of the overall intervention which is conveniently overlooked in favor of the PF plots in the paper conclusions.**

- The purpose of the 24-hour records was exactly as quoted above, and advice based on the record was an important part of the study design. Please note that analysis of all dietary records was provided in the form of a PF plot, and advice was based on the PF plot and aimed at enhancing informed decisions by participants.
- The above points were clarified in Study Design (line 138-141) as well as Discussion (line 352-356).

**2) “The dietary flexibility, self-selection of foods, and no need for daily calorie counting likely contributed to the low attrition.” (434-435)
Data do not support this. Once again with no comparison, it is impossible to know. The study design was very intensive and personal and more likely a contributor to high retention.**

- The sentence is revised to reflect our results more accurately (line 393-394)

**If the study is a feasibility study and the main focus is on the PF plot, then there should have been an evaluation of this plot and how it was received, used, evaluated etc. Yet, questions in the Exit Survey do not ask anything about this. The authors failed to address this concern from the previous review. It remains difficult to determine the relevance of this core component in the absence of a comparator group and any thorough evaluation of these components**.

- Thank you for raising a good point. In the exit survey, we asked about only our overall approach. In retrospect, it would have been more informative if we had added questions on specific components as well.
- That said, because the PF plot was shown to enhance informed decision-making in a restaurant setting, and this entire study was an application of the PF plot to create a self-guided weight loss diet, the outcome of significant weight loss is sufficient to conclude the PF plot-based weight loss is feasible (as clarified in the responses above) without “a comparator group” (addressed in the response to the editor and introduction) or more specific exit survey questions (an optional, not essential component).

**Minor**
**SI Appendix 5: Blood pressure was measured according to the original protocol, why is this not presented?**

- We failed to measure blood pressure throughout the study.

**It is unclear what this means: “Registration was delayed due to lack of knowledge.”**

- We apologize for the confusion. We restated to make the statement clear in line 93-94.

**Reviewer #3: The authors have addressed the comments satisfactorily. I believe that calling the study "individualized diet..." sounds misleading because it appears to refer to personalized nutrition. While not a good term, it seems like it should be called "self-guided diet individualization..."**

- Thank you for your helpful suggestion. The expression “individualized diet” in some parts may be misleading. We edited the “individualized diet” to “self-guided” in line 24.
