# Supplementary material for: A feasibility study to test a novel approach to dietary weight loss with a focus on assisting informed decision making in food selection
Source: PLoS One. 2022 May 26;17(5):e0267876. doi: 10.1371/journal.pone.0267876 (PMC9135285; doi:10.1371/journal.pone.0267876)
Supplement: Peer review history

# Peer review: Decision Letter 0

**DOI:** 10.1371/journal.pone.0267876.r001

**Authors:** Jamie I. Baum

21 Oct 2020

PONE-D-20-14863

Individualized Diet Improvement Program (iDip), an approach to weight loss by combining self-experimenting and self-monitoring using visual feedback: before and after study design

PLOS ONE

Dear Dr. Lee,

Thank you for submitting your manuscript to PLOS ONE. After careful consideration, we feel that it has merit but does not fully meet PLOS ONE’s publication criteria as it currently stands. Therefore, we invite you to submit a revised version of the manuscript that addresses the points raised during the review process.

Be sure to take reviewer comments into consideration before resubmission.

Please submit your revised manuscript by Dec 05 2020 11:59PM. If you will need more time than this to complete your revisions, please reply to this message or contact the journal office at plosone@plos.org. When you're ready to submit your revision, log on to https://www.editorialmanager.com/pone/ and select the 'Submissions Needing Revision' folder to locate your manuscript file.

Please include the following items when submitting your revised manuscript:

- A rebuttal letter that responds to each point raised by the academic editor and reviewer(s). You should upload this letter as a separate file labeled 'Response to Reviewers'.
- A marked-up copy of your manuscript that highlights changes made to the original version. You should upload this as a separate file labeled 'Revised Manuscript with Track Changes'.
- An unmarked version of your revised paper without tracked changes. You should upload this as a separate file labeled 'Manuscript'.

If you would like to make changes to your financial disclosure, please include your updated statement in your cover letter. Guidelines for resubmitting your figure files are available below the reviewer comments at the end of this letter.

If applicable, we recommend that you deposit your laboratory protocols in protocols.io to enhance the reproducibility of your results. Protocols.io assigns your protocol its own identifier (DOI) so that it can be cited independently in the future. For instructions see: http://journals.plos.org/plosone/s/submission-guidelines#loc-laboratory-protocols

We look forward to receiving your revised manuscript.

Kind regards,

Jamie I. Baum, PhD

Academic Editor

PLOS ONE

Additional Editor Comments:

The title of the manuscript inaccurately describes the study. The paper needs the be re-written for syntax.

ABSTRACT

The abstract methodology is vague. What makes this study individualized as described in the title? What was the breakdown of male and female participants and average age? The results are descriptive, with no mention of p-values. Strong conclusions for n=12 finishing the iDip program.

INTRODUCTION

Lines 44-45: These are outdated programs that do not have as large of following as the did when first launched and the authors should include updated diet programs as examples.

Several weight loss/energy reduction methods are described in the introduction. However, the title describes iDip as an individualized program and the introduction should address the importance of addressing individualized needs for successful weight loss.

MATERIALS AND METHODS

Lines 88-132 should come after participant recruitment and study design. This section is actually part of study/intervention design.

-CONSORT guidelines should be followed

-Were medications, alcohol intake, or tobacco use taken into consideration for recruitment?

-No clear power analysis is presented.

-Line 150- reference needed for attrition.

-It is unclear how this study was individualized apart from the 3, 30-minute session offered over the 12 month intervention.

Line 164: be specific beyond periodic. If the objective was to direct food intake based on protein and fiber density, then this feedback seems like it should be more regular than periodic.

Line 183: again, be specific about periodic

How often were participants providing feedback on home weighing

The fiber and protein density plots seem difficult for the lay-person to understand. How was comprehension amongst participants assessed?

How were FFQ collected? Digitally?

RESULTS

Exit survey could become supplemental material

DISCUSSION

-It is unclear how iDip is innovative.

Journal Requirements:

When submitting your revision, we need you to address these additional requirements.

1. Please ensure that your manuscript meets PLOS ONE's style requirements, including those for file naming. The PLOS ONE style templates can be found at

https://journals.plos.org/plosone/s/file?id=wjVg/PLOSOne_formatting_sample_main_body.pdf and

https://journals.plos.org/plosone/s/file?id=ba62/PLOSOne_formatting_sample_title_authors_affiliations.pdf

2. In your Methods section, please provide additional information regarding some discrepancies found  between your submission and the clinical trial protocol. For example, we note that the sample size in the protocol is reported to be 180; in the CT.gov registry, it is mentioned that 44 participants were recruited; while in the manuscript text, the sample size is 15. Please explain these discrepancies, and provide more detail on the how the sample size was calculated. Furthermore, we note that, according to the protocol, the  dietary intake assessment  should have been carried out at 0, 6 and 12 months; while in the manuscript, it is reported that it was carried out only at baseline and at 12 months. Also, please clearly state the dates for participant recruitment and follow-up.

3. Please include additional information regarding the survey or questionnaire used in the study and ensure that you have provided sufficient details that others could replicate the analyses. For instance, if you developed a questionnaire as part of this study and it is not under a copyright more restrictive than CC-BY, please include a copy, in both the original language and English, as Supporting Information. Moreover, please include more details on how the questionnaire was pre-tested, and whether it was validated.

4. Please note that according to our submission guidelines (http://journals.plos.org/plosone/s/submission-guidelines), outmoded terms and potentially stigmatizing labels should be changed to more current, acceptable terminology. For example: “Caucasian” should be changed to “white” or “of [Western] European descent” (as appropriate).

5. Thank you for submitting your clinical trial to PLOS ONE and for providing the name of the registry and the registration number. The information in the registry entry suggests that your trial was registered after patient recruitment began. PLOS ONE strongly encourages authors to register all trials before recruiting the first participant in a study.

As per the journal’s editorial policy, please include in the Methods section of your paper:

1) your reasons for your delay in registering this study (after enrolment of participants started);

2) confirmation that all related trials are registered by stating: “The authors confirm that all ongoing and related trials for this drug/intervention are registered”.

Please also ensure you report the date at which the ethics committee approved the study as well as the complete date range for patient recruitment and follow-up in the Methods section of your manuscript.

6. We note that you have stated that you will provide repository information for your data at acceptance. Should your manuscript be accepted for publication, we will hold it until you provide the relevant accession numbers or DOIs necessary to access your data. If you wish to make changes to your Data Availability statement, please describe these changes in your cover letter and we will update your Data Availability statement to reflect the information you provide.

[Note: HTML markup is below. Please do not edit.]

Reviewers' comments:

Reviewer's Responses to Questions

**Comments to the Author**

1. Is the manuscript technically sound, and do the data support the conclusions?

The manuscript must describe a technically sound piece of scientific research with data that supports the conclusions. Experiments must have been conducted rigorously, with appropriate controls, replication, and sample sizes. The conclusions must be drawn appropriately based on the data presented. 

Reviewer #1: Partly

Reviewer #2: No

**********

2. Has the statistical analysis been performed appropriately and rigorously? 

Reviewer #1: No

Reviewer #2: Yes

**********

3. Have the authors made all data underlying the findings in their manuscript fully available?

The PLOS Data policy requires authors to make all data underlying the findings described in their manuscript fully available without restriction, with rare exception (please refer to the Data Availability Statement in the manuscript PDF file). The data should be provided as part of the manuscript or its supporting information, or deposited to a public repository. For example, in addition to summary statistics, the data points behind means, medians and variance measures should be available. If there are restrictions on publicly sharing data—e.g. participant privacy or use of data from a third party—those must be specified.

Reviewer #1: No

Reviewer #2: Yes

**********

4. Is the manuscript presented in an intelligible fashion and written in standard English?

PLOS ONE does not copyedit accepted manuscripts, so the language in submitted articles must be clear, correct, and unambiguous. Any typographical or grammatical errors should be corrected at revision, so please note any specific errors here.

Reviewer #1: Yes

Reviewer #2: Yes

**********

5. Review Comments to the Author

Please use the space provided to explain your answers to the questions above. You may also include additional comments for the author, including concerns about dual publication, research ethics, or publication ethics. (Please upload your review as an attachment if it exceeds 20,000 characters)

Reviewer #1: The title is too long and should reflect the fact that it is a pilot study to test the feasibility of iDip on weight loss at 12 months.

The initial protocol stated that, 30 participants would be in an intervention group and 30 in the wait-list group. The study design reported in this paper involved only 12 participants in a before-after comparison. Please explain. Rather than using the minimum sample size reported in other published studies, a formal power calculation should be provided to support the selected sample size. With more than one primary outcomes, what was the minimum effect size to detect post intervention?

Data analysis only considered the paired t-tests on the differences before and after the intervention, and Pearson correlation on the associations between body weight and protein/fiber density. For a small sample of 12 participants who completed the intervention, have the authors checked the distribution of outcomes and considered non-parametric tests? The authors also compared the two groups of participants between the top and bottom half in weight loss at 12 months, what test was performed here? The repeated measures ANOVA reported in Figure 7 should also be mentioned in statistical analysis section.

As this is not a randomised trial, baseline demographics and medical histories can be reported in Table 1 on the 12 participants who completed the intervention at 12 months. The two participants that discontinued the intervention can be described separately in text. It is also important to present baseline characteristics of the two groups of participants above or below 5% weight loss at 12 months and see how they may differ.

The raw outcomes presented in Table 2 would be better presented as a full dataset at baseline and 12 months, since the primary and secondary outcomes were measured at these two time points on a total of 12 participants.

There was no Table 3. For Table 4, I would suggest reporting median together with mean (SD) for a score of 1-7. The SE is redundant and should be removed.

Both Pearson and Spearman correlation coefficients should be reported in a small sample.

Note that mean weight loss is different from % weight loss, and the terms should not be used interchangeably.

Reviewer #2: This paper reports on a small study evaluating the Individualized Diet Improvement Program (iDip) at 12 months. The 14 participants are only evaluated pre and post.

Major

1. Study design

This study lacks a comparison group which makes the authors' claims about the success of the program and the critical role of certain aspects of the program (the plot) unfounded. This evaluation is also only completed on a small group of people. If the sample were small, but the study design more rigorous, this alone would not be a major issue. However, in combination, it severely impairs any contribution to scientific knowledge.

2. Intervention

It is unclear what exactly is novel and innovative about this weight management approach. The Protein-Fiber plot seems to be a big part of it. There is no evaluation of whether this plot was really used and all participants received it, so it is impossible to know its actual contribution to the program. The plot itself also appears to present only single foods and does not seem to account for whole diet or mixed foods (like casseroles) which is a major limitation.

In the discussion, the study is described as innovative because it does not use calorie counting, specific food advice and is non-restrictive. But, ultimately the program applies a 25% energy restriction. In the discussion it says that participants were "encouraged to include all foods and self-select to moderate intake of foods with low protein and fiber densities". Given that the program included 19, 60-min meetings and 3 individual sessions delivered by a registered dietician, it would appear that this program is little more than a group-based, dietitian program.

Despite suggestion that this program lacked detailed food or intake monitoring, the participants did monitor their food intake by completing 6 different 24h recalls. Although not a formal part of the program, with no comparator, it is impossible to know the effects of this. Previous studies have shown the importance of monitoring and how it alone can reduce intake.

Minor

1. Why does the program include daily weighing? This can be a controversial recommendation that can result in obsessing over insignificant changes.

2. Figure 8 is an R output that may not be easily interpretable by many readers.

3. In the method, it states that dietary recalls were "periodically" completed. This needs to be specified.

4. The intervention and its specific novelty is not made particularly clear. Parts of the discussion provide better insight than the method, but this should be clear long before

5. The expenditure side of energy balance is not acknowledged in the introduction.

6. The discussion describes the diet as non-restrictive. It includes a 25% restriction.

7. There is no citation for the Power calculation.

8. There may exist an opportunity to explore how participants chose to restrict their diet and whether certain patterns were more successful. This could be more interesting as an outcome of the study and more of a contribution to the field.

**********

6. PLOS authors have the option to publish the peer review history of their article (what does this mean?). If published, this will include your full peer review and any attached files.

If you choose “no”, your identity will remain anonymous but your review may still be made public.

**Do you want your identity to be public for this peer review?** For information about this choice, including consent withdrawal, please see our Privacy Policy.

Reviewer #1: No

Reviewer #2: No

[NOTE: If reviewer comments were submitted as an attachment file, they will be attached to this email and accessible via the submission site. Please log into your account, locate the manuscript record, and check for the action link "View Attachments". If this link does not appear, there are no attachment files.]

While revising your submission, please upload your figure files to the Preflight Analysis and Conversion Engine (PACE) digital diagnostic tool, https://pacev2.apexcovantage.com/. PACE helps ensure that figures meet PLOS requirements. To use PACE, you must first register as a user. Registration is free. Then, login and navigate to the UPLOAD tab, where you will find detailed instructions on how to use the tool. If you encounter any issues or have any questions when using PACE, please email PLOS at figures@plos.org. Please note that Supporting Information files do not need this step.

# Author comment: Author response to Decision Letter 0

**DOI:** 10.1371/journal.pone.0267876.r002

4 Dec 2020

Thank you for your careful evaluations of our manuscript and helpful comments. As shown below, we addressed all of the comments and revised the manuscript accordingly. With this revision, we believe that descriptions of the study are clearer, and the manuscript is easier to follow.

Additional Editor Comments:

The title of the manuscript inaccurately describes the study. The paper needs the be re-written for syntax.

• Title has been revised to better summarize the study.

ABSTRACT

The abstract methodology is vague. What makes this study individualized as described in the title? What was the breakdown of male and female participants and average age? The results are descriptive, with no mention of p-values. Strong conclusions for n=12 finishing the iDip program.

• Clarification of individualization: In lines 5-8, the abstract has been reworded to emphasize the self-selected dietary changes by individual participants.

• Average age and gender proportion of the participants are included in line 15.

• P-values are added for results.

• The conclusion relates only to the feasibility of the approach and does not make claims beyond that.

INTRODUCTION

Lines 44-45: These are outdated programs that do not have as large of following as the did when first launched and the authors should include updated diet programs as examples.

• Excluding certain food groups are a mainstay of dieting books although popularity may come and go. Atkins and Zone diets are still used by many. More importantly the new and trendy diets are often rebranding of old diet programs. For example, the keto diet, which is currently very popular, is essentially the same as the Atkins diet although it is marketed as novel. The paleo and plant-based diets are similar to Zone and Ornish diets, respectively. This point is added to the Introduction in lines 43-48.

Several weight loss/energy reduction methods are described in the introduction. However, the title describes iDip as an individualized program and the introduction should address the importance of addressing individualized needs for successful weight loss.

• The last paragraph (line 80-94) of the Introduction is revised to highlight the individualized approach of our study.

MATERIALS AND METHODS

Lines 88-132 should come after participant recruitment and study design. This section is actually part of study/intervention design.

• This section has moved to line 146-191.

CONSORT guidelines should be followed

• After confirming with the PLOS ONE editorial office, the TREND checklist should be used due to the non-randomized study design.

Were medications, alcohol intake, or tobacco use taken into consideration for recruitment?

• Medications (except for insulin), alcohol intake, or tobacco use were not taken into consideration for recruitment as described in Participant recruitment.

No clear power analysis is presented.

• Details of power calculation to determine the sample size were added to lines 106-113.

Line 150- reference needed for attrition.

• Reference is added in line 112.

It is unclear how this study was individualized apart from the 3, 30-minute session offered over the 12 month intervention.

• What we meant by “individualized” was that individuals decide and create their weight loss diet, not that instructors provide several one-on-one advising sessions. This point is clarified in both the Abstract (Line 5-8) and Introduction (Line 82-90).

Line 164: be specific beyond periodic. If the objective was to direct food intake based on protein and fiber density, then this feedback seems like it should be more regular than periodic.

• Timepoints were added in line 141.

Line 183: again, be specific about periodic. 

• Timepoints were added in lines 208-209.

How often were participants providing feedback on home weighing?

• The weight chart was provided to participants on a weekly basis as mentioned in line 140.

The fiber and protein density plots seem difficult for the lay-person to understand. How was comprehension amongst participants assessed?

• Based on “Improvements in recall and food choices using a graphical method to deliver information of select nutrients,” reported by Pratt et al., the comprehension of the plot was high as 83.8% of participants (n=142) responded that they understood the nutrition graph. 

• Our participants also reported the visual feedbacks were easy to understand and helpful as mentioned in lines 331-332.

How were FFQ collected? Digitally?

• The FFQ was collected in a paper version and we clarified this point in lines 205-206. 

RESULTS

Exit survey could become supplemental material

• Exit survey was moved to the supplemental materials, as S3 Appendix.

DISCUSSION

It is unclear how iDip is innovative.

• We have revised the Abstract (lines 5-8), Introduction (43-48, 56-59, 80-94), Methods (146-191) to clearly state our innovative approach in the early part of the manuscript.

Journal Requirements:

When submitting your revision, we need you to address these additional requirements.

1. Please ensure that your manuscript meets PLOS ONE's style requirements, including those for file naming. The PLOS ONE style templates can be found at

https://journals.plos.org/plosone/s/file?id=wjVg/PLOSOne_formatting_sample_main_body.pdf and

https://journals.plos.org/plosone/s/file?id=ba62/PLOSOne_formatting_sample_title_authors_affiliations.pdf

2. In your Methods section, please provide additional information regarding some discrepancies found between your submission and the clinical trial protocol. For example, we note that the sample size in the protocol is reported to be 180; in the CT.gov registry, it is mentioned that 44 participants were recruited; while in the manuscript text, the sample size is 15. Please explain these discrepancies, and provide more detail on the how the sample size was calculated. Furthermore, we note that, according to the protocol, the dietary intake assessment should have been carried out at 0, 6 and 12 months; while in the manuscript, it is reported that it was carried out only at baseline and at 12 months. Also, please clearly state the dates for participant recruitment and follow-up.

• IRB approved the iDip as a part of a series of studies.

• The registry has been revised to publish each trial separately. The first study described in this manuscript is now registered with 14 participants.

• Dates are specified in lines 96-102.

3. Please include additional information regarding the survey or questionnaire used in the study and ensure that you have provided sufficient details that others could replicate the analyses. For instance, if you developed a questionnaire as part of this study and it is not under a copyright more restrictive than CC-BY, please include a copy, in both the original language and English, as Supporting Information. Moreover, please include more details on how the questionnaire was pre-tested, and whether it was validated.

• We used a validated FFQ (S2_Appendix) as shown in lines 205-208.

• We appreciate your kind suggestion. Exit survey is now S3_Appendix.

4. Please note that according to our submission guidelines (http://journals.plos.org/plosone/s/submission-guidelines), outmoded terms and potentially stigmatizing labels should be changed to more current, acceptable terminology. For example: “Caucasian” should be changed to “white” or “of [Western] European descent” (as appropriate).

• In Table 1, we have revised to White from Caucasian. 

5. Thank you for submitting your clinical trial to PLOS ONE and for providing the name of the registry and the registration number. The information in the registry entry suggests that your trial was registered after patient recruitment began. PLOS ONE strongly encourages authors to register all trials before recruiting the first participant in a study.

As per the journal’s editorial policy, please include in the Methods section of your paper:

1) your reasons for your delay in registering this study (after enrolment of participants started); 

• We mentioned our reason for our delay in lines 98-99.

2) confirmation that all related trials are registered by stating: “The authors confirm that all ongoing and related trials for this drug/intervention are registered”.

• We made a confirmation in lines 99-100.

Please also ensure you report the date at which the ethics committee approved the study as well as the complete date range for patient recruitment and follow-up in the Methods section of your manuscript.

• The report date at which the ethics committee approved and the complete data range for recruitment and follow-up are stated in lines 97, 100-101.

6. We note that you have stated that you will provide repository information for your data at acceptance. Should your manuscript be accepted for publication, we will hold it until you provide the relevant accession numbers or DOIs necessary to access your data. If you wish to make changes to your Data Availability statement, please describe these changes in your cover letter and we will update your Data Availability statement to reflect the information you provide.

• DOI is 10.13012/B2IDB-4710255_V1.

Reviewer #1: 

The title is too long and should reflect the fact that it is a pilot study to test the feasibility of iDip on weight loss at 12 months.

• Removed Individualized Dietary Improvement Program (iDip) in title and added “Feasibility”.

The initial protocol stated that, 30 participants would be in an intervention group and 30 in the wait-list group. The study design reported in this paper involved only 12 participants in a before-after comparison. Please explain. 

• Because of the feasibility nature of the study and logistic limitations, we started with half the size of original plan, n=14 instead of n~30, and planned to repeat the procedure if necessary. However, we were able to achieve the primary objective with the first cohort. Thus, rather than repeating the same trial, we amended the IRB protocol for the second trial, which is currently ongoing. Also, because the number of wait-listed participants declined in the process, we had to adopt the before and after design instead.

Rather than using the minimum sample size reported in other published studies, a formal power calculation should be provided to support the selected sample size. With more than one primary outcomes, what was the minimum effect size to detect post intervention?

• Details of the power calculation to determine the sample size were added to lines 106-113.

Data analysis only considered the paired t-tests on the differences before and after the intervention, and Pearson correlation on the associations between body weight and protein/fiber density. For a small sample of 12 participants who completed the intervention, have the authors checked the distribution of outcomes and considered non-parametric tests? The authors also compared the two groups of participants between the top and bottom half in weight loss at 12 months, what test was performed here? The repeated measures ANOVA reported in Figure 7 should also be mentioned in statistical analysis section.

• Lines 226-228 are added to show enhanced detail on statistical analyses including distribution of outcomes. 

• We did not consider non-parametric tests because Shapiro-Wilk testing indicated our data set was normal. 

• Top and bottom groups were divided into two halves by the magnitude of weight loss. No statistics should be applied between their weights. We did run statistics on diet between the two groups (Fig 7). As mentioned in the captions, we used paired t-test.

• The repeated measures ANOVA is added in the methods section.

As this is not a randomised trial, baseline demographics and medical histories can be reported in Table 1 on the 12 participants who completed the intervention at 12 months. The two participants that discontinued the intervention can be described separately in text. It is also important to present baseline characteristics of the two groups of participants above or below 5% weight loss at 12 months and see how they may differ.

• We added another column to show the characteristics of completers in Table 1.

• No significance was found in any demographic data between the top and bottom groups in weight loss. A sentence is added to Results in lines 242-243.

The raw outcomes presented in Table 2 would be better presented as a full dataset at baseline and 12 months, since the primary and secondary outcomes were measured at these two time points on a total of 12 participants.

• We believe adding 6 and 18 month data is informative.

There was no Table 3. For Table 4, I would suggest reporting median together with mean (SD) for a score of 1-7. The SE is redundant and should be removed.

• We apologize for our mistake and appreciate your pointing this out. We have changed Table 4 to Table 3 and eliminated SE. Table 3 has become S3 Appendix.

Both Pearson and Spearman correlation coefficients should be reported in a small sample.

• Because Shapiro-Wilk testing indicated our sample, while small, was normal. As such, we do not find it necessary to utilize non-parametric testing. 

Note that mean weight loss is different from % weight loss, and the terms should not be used interchangeably.

• Revisions have been made throughout the manuscript to not to use these terms interchangeably. 

Reviewer #2: 

This paper reports on a small study evaluating the Individualized Diet Improvement Program (iDip) at 12 months. The 14 participants are only evaluated pre and post.

Major

1. Study design

This study lacks a comparison group which makes the authors' claims about the success of the program and the critical role of certain aspects of the program (the plot) unfounded. This evaluation is also only completed on a small group of people. If the sample were small, but the study design more rigorous, this alone would not be a major issue. However, in combination, it severely impairs any contribution to scientific knowledge.

• This is a feasibility study to test if the novel, dietary approach can deliver a mean weight loss of >5% initial body weight. A before-and-after design without a control group is frequently used for weight loss studies of 12 months or less because it is very unlikely that a control group achieves >5% weight loss without any intervention in this time span.

• Efficacy of the PF plot for a food selection by customers in a restaurant setting was demonstrated in our previous study “Improvements in recall and food choices using a graphical method to deliver information of select nutrients,” reported by Pratt et al. Applying the PF plot in achieving weight loss was part of the study objectives, but we did not find any overstatement of our study results regarding the PF plot’s efficacy in our manuscript. Based on the editor and the other reviewer’s comments, we revised to present the innovative nature of our approach including the PF plot more clearly in Abstract (lines 5-8) and Introduction (lines 82-90).

• Details of the power calculation to determine the sample size were added to lines 106-113.

2. Intervention

It is unclear what exactly is novel and innovative about this weight management approach. The Protein-Fiber plot seems to be a big part of it. There is no evaluation of whether this plot was really used and all participants received it, so it is impossible to know its actual contribution to the program. The plot itself also appears to present only single foods and does not seem to account for whole diet or mixed foods (like casseroles) which is a major limitation. 

• As mentioned in the reply to the previous query, the innovative approach is better highlighted throughout the revised manuscript

• PF plot is the only analysis and feedback on participants’ diet. Participants were able to create and advance their weight loss diet based on the plot. No single recipe, food exclusions, or meal plans were provided to them. This point is clarified in the Methods section in lines 142-143 and 350-351.

• As shown in Figures one, five and six as well as Pratt et al 2016, the advantage of the PF plot is it can plot individual foods, a dish (like casserole), meal, or meals. Alternatively, a dish can be plotted by individual ingredients as well to improve a recipe. In a previous study (Pratt et al), there were significant improvements in cafeteria food choices when the plot was displayed, whereas the presence of a nutrition facts label had no effect. Additionally, the blue total dot, as mentioned in Figure one caption, displays the meal or day’s average nutritional value.

In the discussion, the study is described as innovative because it does not use calorie counting, specific food advice and is non-restrictive. But, ultimately the program applies a 25% energy restriction. 

• The expression “non-restrictive” may be misleading. We replaced it with “no exclusion of certain food groups.” This point is clarified by rewording in lines 405 and 414. 

• We made it clear to the participants that their daily energy intake must be reduced to achieve weight loss. This is the rationale for the two target boxes for weight maintenance and weight loss in the PF plot (Figure one and lines 163-168). However, the participants reduced the energy intake by choosing from green foods in the PF plot to move their meal closer to the target weight loss box. Energy balance was monitored via daily self-weighing. The difference is that participants achieved negative energy balance without traditional daily calorie counting or following portion controlled daily menus. 

In the discussion it says that participants were "encouraged to include all foods and self-select to moderate intake of foods with low (high) protein and fiber densities". 

• We rephrased it to make our point clear as follows: “Participants were encouraged to self-select to limit intake of foods that contain minimal fiber and protein, but they were not instructed to exclude any particular foods from their diet.” (lines 358-360) 

Given that the program included 19, 60-min meetings and 3 individual sessions delivered by a registered dietician, it would appear that this program is little more than a group-based, dietitian program.

• We assume the reviewer is questioning the individualized nature of our program. What we meant by “individualized” was that individuals decide and create their weight loss diet, not that instructors provide several one-on-one advising sessions. This point is clarified in Abstract (lines 5-8) and Introduction (lines 82-90).

Despite suggestion that this program lacked detailed food or intake monitoring, the participants did monitor their food intake by completing 6 different 24h recalls. Although not a formal part of the program, with no comparator, it is impossible to know the effects of this. Previous studies have shown the importance of monitoring and how it alone can reduce intake.

• The participants achieved significant weight loss with only six 24-hour records in the 12-month period and daily weighing (this is a daily monitoring although not food intake monitoring). An important difference is time required for participants for 6 24-hour records and daily weighing was much less than daily food intake monitoring. Previous studies did show monitoring alone could reduce intake. However, to our knowledge, there is no study that reported weight loss success comparable to ours in magnitude, duration, and retention by daily food intake monitoring alone.

Minor

1. Why does the program include daily weighing? This can be a controversial recommendation that can result in obsessing over insignificant changes.

• As stated in lines 178-184, daily weighing is the easiest and fastest way to monitor energy balance. “Frequent self-weighing with electronic graphic feedback to prevent age-related weight gain in young adults,” by Bertz et al. has shown that providing weekly feedback prevents weight gain in young adults. Daily weighing shows the fluctuations but more importantly displays an overall trend. We emphasized that participants should look at weekly and monthly trends, not daily fluctuations. Our participants reported no problems with daily weighing. 

2. Figure 8 is an R output that may not be easily interpretable by many readers.

• The matrix was used in Figure eight as it displays relatively minor findings and saves space.

3. In the method, it states that dietary recalls were "periodically" completed. This needs to be specified.

• The months were data was collected are specified in the legend of Figure seven, and also added to the methods (lines 141 and 208-209).

4. The intervention and its specific novelty is not made particularly clear. Parts of the discussion provide better insight than the method, but this should be clear long before

• Thank you for your helpful comment. We have revised the Abstract (lines 5-8), Introduction (43-48, 56-59, 80-94), Methods (146-191) to state our innovative approach clearly in the early part of the manuscript.

5. The expenditure side of energy balance is not acknowledged in the introduction.

• If the “expenditure side of energy balance” refers to exercise, it is very challenging to achieve the large negative energy balance necessary for weight loss via physical activity only. As such, we focused on the dietary aspect of weight loss in our program.

6. The discussion describes the diet as non-restrictive. It includes a 25% restriction.

• What we meant by “non-restrictive” was not to exclude any food groups from diet. This point is clarified by changing wording in lines 405 and 414.

7. There is no citation for the Power calculation.

• The details of power calculation to determine the sample size were added to lines 106-113.

8. There may exist an opportunity to explore how participants chose to restrict their diet and whether certain patterns were more successful. This could be more interesting as an outcome of the study and more of a contribution to the field.

• Thank you for an interesting suggestion. We will explore it in future, larger studies.

Attachment Submitted filename: Response to Reviewers.docx Click here for additional data file.

# Peer review: Decision Letter 1

**DOI:** 10.1371/journal.pone.0267876.r003

**Authors:** John W. Apolzan

9 Aug 2021

PONE-D-20-14863R1

A feasibility study to test a novel approach to dietary weight loss with a focus on assisting informed decision making in food selection

PLOS ONE

Dear Dr. Lee,

Thank you for submitting your manuscript to PLOS ONE. After careful consideration, we feel that it has merit but does not fully meet PLOS ONE’s publication criteria as it currently stands. Therefore, we invite you to submit a revised version of the manuscript that addresses the points raised during the review process.

Please submit your revised manuscript by Sep 23 2021 11:59PM. If you will need more time than this to complete your revisions, please reply to this message or contact the journal office at plosone@plos.org. When you're ready to submit your revision, log on to https://www.editorialmanager.com/pone/ and select the 'Submissions Needing Revision' folder to locate your manuscript file.

Please include the following items when submitting your revised manuscript:

- A rebuttal letter that responds to each point raised by the academic editor and reviewer(s). You should upload this letter as a separate file labeled 'Response to Reviewers'.
- A marked-up copy of your manuscript that highlights changes made to the original version. You should upload this as a separate file labeled 'Revised Manuscript with Track Changes'.
- An unmarked version of your revised paper without tracked changes. You should upload this as a separate file labeled 'Manuscript'.

If you would like to make changes to your financial disclosure, please include your updated statement in your cover letter. Guidelines for resubmitting your figure files are available below the reviewer comments at the end of this letter.

If applicable, we recommend that you deposit your laboratory protocols in protocols.io to enhance the reproducibility of your results. Protocols.io assigns your protocol its own identifier (DOI) so that it can be cited independently in the future. For instructions see: http://journals.plos.org/plosone/s/submission-guidelines#loc-laboratory-protocols. Additionally, PLOS ONE offers an option for publishing peer-reviewed Lab Protocol articles, which describe protocols hosted on protocols.io. Read more information on sharing protocols at https://plos.org/protocols?utm_medium=editorial-email&utm_source=authorletters&utm_campaign=protocols.

We look forward to receiving your revised manuscript.

Kind regards,

John W. Apolzan, PhD

Academic Editor

PLOS ONE

Additional Editor Comments (if provided):

As a reviewer notes, the tone of the manuscript needs to ensure that it is a feasibility study without a control group. The lack of a control group is a study limitation that needs to be stated. Please ensure the reviewer responses are addressed. Use of terms such as non-randomized and single arm trial would be beneficial throughout the manuscript including the conclusions. Further, the pilot or feasibility consort should be utilized. Also, the (TIDieR) checklist and guide should be used to describe the intervention.

[Note: HTML markup is below. Please do not edit.]

Reviewers' comments:

Reviewer's Responses to Questions

**Comments to the Author**

1. If the authors have adequately addressed your comments raised in a previous round of review and you feel that this manuscript is now acceptable for publication, you may indicate that here to bypass the “Comments to the Author” section, enter your conflict of interest statement in the “Confidential to Editor” section, and submit your "Accept" recommendation.

Reviewer #2: (No Response)

Reviewer #3: (No Response)

**********

2. Is the manuscript technically sound, and do the data support the conclusions?

The manuscript must describe a technically sound piece of scientific research with data that supports the conclusions. Experiments must have been conducted rigorously, with appropriate controls, replication, and sample sizes. The conclusions must be drawn appropriately based on the data presented. 

Reviewer #2: No

Reviewer #3: Yes

**********

3. Has the statistical analysis been performed appropriately and rigorously? 

Reviewer #2: Yes

Reviewer #3: Yes

**********

4. Have the authors made all data underlying the findings in their manuscript fully available?

The PLOS Data policy requires authors to make all data underlying the findings described in their manuscript fully available without restriction, with rare exception (please refer to the Data Availability Statement in the manuscript PDF file). The data should be provided as part of the manuscript or its supporting information, or deposited to a public repository. For example, in addition to summary statistics, the data points behind means, medians and variance measures should be available. If there are restrictions on publicly sharing data—e.g. participant privacy or use of data from a third party—those must be specified.

Reviewer #2: Yes

Reviewer #3: Yes

**********

5. Is the manuscript presented in an intelligible fashion and written in standard English?

PLOS ONE does not copyedit accepted manuscripts, so the language in submitted articles must be clear, correct, and unambiguous. Any typographical or grammatical errors should be corrected at revision, so please note any specific errors here.

Reviewer #2: Yes

Reviewer #3: Yes

**********

6. Review Comments to the Author

Please use the space provided to explain your answers to the questions above. You may also include additional comments for the author, including concerns about dual publication, research ethics, or publication ethics. (Please upload your review as an attachment if it exceeds 20,000 characters)

Reviewer #2: Major

Issues raised about the study design previously remain. Without a control group, the authors cannot conclude that the PF plot has efficacy over and above the methodological aspects of the study which included an extensive amount of dietary recall and FFQ and dietary coaching with a health professional. Further, as a feasibility study, it lacks any in-depth evaluation of the innovative components of the intervention (eg, the PF plots).

The authors state that they could not find any overstatement in their conclusions. Two concrete examples are provided below. Each shows a lack of acknowledgement of study limitations while also being worded too strongly.

1) “The individualized PF plot played a critical role in dietary improvement and allowed participants to use the knowledge gained from education sessions to self select foods to increase or decrease.”

Regular and intensive dietary recall and meetings with a dietician were much more likely the cause of this and the study design does not allow these to be teased apart. There is no evaluation of the possible mechanisms either. This statement suggests a linear process that has been demonstrated. The study provides no data to support this strongly worded statement. Furthermore, the ethics application notes that the purpose of the dietary recall is to “check adequacy of their diet and to troubleshoot if a participant is not losing weight effectively” so the recall process is seemingly a significant part of the overall intervention which is conveniently overlooked in favor of the PF plots in the paper conclusions.

2) “The dietary flexibility, self-selection of foods, and no need for daily calorie counting likely contributed to the low attrition.”

Data do not support this. Once again with no comparison, it is impossible to know. The study design was very intensive and personal and more likely a contributor to high retention.

If the study is a feasibility study and the main focus is on the PF plot, then there should have been an evaluation of this plot and how it was received, used, evaluated etc. Yet, questions in the Exit Survey do not ask anything about this. The authors failed to address this concern from the previous review. It remains difficult to determine the relevance of this core component in the absence of a comparator group and any thorough evaluation of these components.

Minor

SI Appendix 5: Blood pressure was measured according to the original protocol, why is this not presented?

It is unclear what this means: “Registration was delayed due to lack of knowledge.”

Reviewer #3: The authors have addressed the comments satisfactorily. I believe that calling the study "individualized diet..." sounds misleading because it appears to refer to personalized nutrition. While not a good term, it seems like it should be called "self-guided diet individualization..."

**********

7. PLOS authors have the option to publish the peer review history of their article (what does this mean?). If published, this will include your full peer review and any attached files.

If you choose “no”, your identity will remain anonymous but your review may still be made public.

**Do you want your identity to be public for this peer review?** For information about this choice, including consent withdrawal, please see our Privacy Policy.

Reviewer #2: No

Reviewer #3: No

[NOTE: If reviewer comments were submitted as an attachment file, they will be attached to this email and accessible via the submission site. Please log into your account, locate the manuscript record, and check for the action link "View Attachments". If this link does not appear, there are no attachment files.]

While revising your submission, please upload your figure files to the Preflight Analysis and Conversion Engine (PACE) digital diagnostic tool, https://pacev2.apexcovantage.com/. PACE helps ensure that figures meet PLOS requirements. To use PACE, you must first register as a user. Registration is free. Then, login and navigate to the UPLOAD tab, where you will find detailed instructions on how to use the tool. If you encounter any issues or have any questions when using PACE, please email PLOS at figures@plos.org. Please note that Supporting Information files do not need this step.

# Author comment: Author response to Decision Letter 1

**DOI:** 10.1371/journal.pone.0267876.r004

3 Sep 2021

Response to Reviewers

We truly appreciate your thoughtful review of our manuscript and helpful comments. As shown below, we addressed the comments and revised the manuscript accordingly. With this revision, we believe that descriptions of the study are clearer, and the manuscript is easier to follow.

Additional Editor Comments:

As a reviewer notes, the tone of the manuscript needs to ensure that it is a feasibility study without a control group. The lack of a control group is a study limitation that needs to be stated. Please ensure the reviewer responses are addressed. Use of terms such as non-randomized and single arm trial would be beneficial throughout the manuscript including the conclusions. 

• Thank you for your kind suggestion. We stated non-randomized and single-arm trial in lines 123, 343, and 433.

• We do not consider the lack of a non-treatment control group as a major limitation of this study. Although we agree that a randomized and controlled trial (RCT) is a gold standard of clinical studies, in the case of weight loss study, a large-scale RCT (DPP Research Group, 2002) has shown that the weight of the placebo group was unchanged after one year, whereas metformin and lifestyle group significantly lost weight. This point was added to the Discussion in line 416-421.

Further, the pilot or feasibility consort should be utilized. Also, the (TIDieR) checklist and guide should be used to describe the intervention.

CONSORT for pilot and feasibility study

• We added the timeline of intervention in line 131-132 to follow the guideline.

• We added the CONSORT as S4 Appendix.

TIDieR 

• We added the objective of the study in line 125-127 and the location of study administration in line 131.

• We added the checklist as S5 Appendix.

Reviewer #2: 

Major

Issues raised about the study design previously remain. Without a control group, the authors cannot conclude that the PF plot has efficacy over and above the methodological aspects of the study which included an extensive amount of dietary recall and FFQ and dietary coaching with a health professional. Further, as a feasibility study, it lacks any in-depth evaluation of the innovative components of the intervention (eg, the PF plots).

• Thank you for raising questions, which seem to be due to unclear descriptions of our objectives and intervention protocol.

• The objective of this study is not to compare the efficacy of our approach with certain conventional approaches. The objective of this feasibility study is to determine if our new approach that replaced conventional methods works. Revisions are made to clarify this point in the revised last paragraph (line 80-87) of Introduction, Study Deign (line 125-127; 129-132; 141-144), and Development of novel learning tools (line 147-151; 169-172) in Methods 

• For this objective, a control group should be a non-treatment group, not a certain traditional weight-loss method. The lack of non-treatment control is addressed in the reply to the editor above.

• Although graduate students with RDN credentials in this research project provided lectures and coaching, the entire interactions in both group sessions and individual advice were performed to test the new approach:

o Feedback of FFQ, 24-hour records were always provided using the PF plot.

o No food was excluded from participant’s diet, and even no recipe was provided to them. As the manuscript title shows, what dietitians provided was a visual comparison of food properties for participants to make informed decisions.

o Efficacy of visualization of nutrient values with PF plot was previously reported (Pratt, 2016).

• These points were stated clearly in the revised Methods (lines 125-127, 137-144, and 147-151) and Discussion (lines 352-366 and 368-381).

• In-depth evaluation of innovative components would be optional because weight loss was set as the primary outcome in our study design. This reviewer’s comment overlaps with the comment on the exit survey, which is addressed below.

The authors state that they could not find any overstatement in their conclusions. Two concrete examples are provided below. Each shows a lack of acknowledgement of study limitations while also being worded too strongly.

1) “The individualized PF plot played a critical role in dietary improvement and allowed participants to use the knowledge gained from education sessions to self select foods to increase or decrease.” (line 404-406)

• The wording of the statement is revised to reflect our results accurately (line 367-368).

Regular and intensive dietary recall and meetings with a dietician were much more likely the cause of this and the study design does not allow these to be teased apart. There is no evaluation of the possible mechanisms either. This statement suggests a linear process that has been demonstrated. The study provides no data to support this strongly worded statement. Furthermore, the ethics application notes that the purpose of the dietary recall is to “check adequacy of their diet and to troubleshoot if a participant is not losing weight effectively” so the recall process is seemingly a significant part of the overall intervention which is conveniently overlooked in favor of the PF plots in the paper conclusions.

• The purpose of the 24-hour records was exactly as quoted above, and advice based on the record was an important part of the study design. Please note that analysis of all dietary records was provided in the form of a PF plot, and advice was based on the PF plot and aimed at enhancing informed decisions by participants. 

• The above points were clarified in Study Design (line 138-141) as well as Discussion (line 352-356).

2) “The dietary flexibility, self-selection of foods, and no need for daily calorie counting likely contributed to the low attrition.” (434-435)

Data do not support this. Once again with no comparison, it is impossible to know. The study design was very intensive and personal and more likely a contributor to high retention. 

• The sentence is revised to reflect our results more accurately (line 393-394).

If the study is a feasibility study and the main focus is on the PF plot, then there should have been an evaluation of this plot and how it was received, used, evaluated etc. Yet, questions in the Exit Survey do not ask anything about this. The authors failed to address this concern from the previous review. It remains difficult to determine the relevance of this core component in the absence of a comparator group and any thorough evaluation of these components.

• Thank you for raising a good point. In the exit survey, we asked about only our overall approach. In retrospect, it would have been more informative if we had added questions on specific components as well. 

• That said, because the PF plot was shown to enhance informed decision-making in a restaurant setting, and this entire study was an application of the PF plot to create a self-guided weight loss diet, the outcome of significant weight loss is sufficient to conclude the PF plot-based weight loss is feasible (as clarified in the responses above) without “a comparator group” (addressed in the response to the editor and introduction) or more specific exit survey questions (an optional, not essential component).

Minor

SI Appendix 5: Blood pressure was measured according to the original protocol, why is this not presented?

• We failed to measure blood pressure throughout the study.

It is unclear what this means: “Registration was delayed due to lack of knowledge.”

• We apologize for the confusion. We restated to make the statement clear in line 93-94.

Reviewer #3: The authors have addressed the comments satisfactorily. I believe that calling the study "individualized diet..." sounds misleading because it appears to refer to personalized nutrition. While not a good term, it seems like it should be called "self-guided diet individualization..."

• Thank you for your helpful suggestion. The expression “individualized diet” in some parts may be misleading. We edited the “individualized diet” to “self-guided” in line 24.

Attachment Submitted filename: Response to Reviewers.docx Click here for additional data file.

# Editor report: Decision Letter 2

**DOI:** 10.1371/journal.pone.0267876.r005

**Authors:** John W. Apolzan

19 Apr 2022

A feasibility study to test a novel approach to dietary weight loss with a focus on assisting informed decision making in food selection

PONE-D-20-14863R2

Dear Dr. Lee,

We’re pleased to inform you that your manuscript has been judged scientifically suitable for publication and will be formally accepted for publication once it meets all outstanding technical requirements.

Within one week, you’ll receive an e-mail detailing the required amendments. When these have been addressed, you’ll receive a formal acceptance letter and your manuscript will be scheduled for publication.

An invoice for payment will follow shortly after the formal acceptance. To ensure an efficient process, please log into Editorial Manager at http://www.editorialmanager.com/pone/, click the 'Update My Information' link at the top of the page, and double check that your user information is up-to-date. If you have any billing related questions, please contact our Author Billing department directly at authorbilling@plos.org.

If your institution or institutions have a press office, please notify them about your upcoming paper to help maximize its impact. If they’ll be preparing press materials, please inform our press team as soon as possible -- no later than 48 hours after receiving the formal acceptance. Your manuscript will remain under strict press embargo until 2 pm Eastern Time on the date of publication. For more information, please contact onepress@plos.org.

Kind regards,

John W. Apolzan, PhD

Academic Editor

PLOS ONE

Additional Editor Comments (optional):

Reviewers' comments:

# Editor report: Acceptance letter

**DOI:** 10.1371/journal.pone.0267876.r006

**Authors:** John W. Apolzan

18 May 2022

PONE-D-20-14863R2 

A feasibility study to test a novel approach to dietary weight loss with a focus on assisting informed decision making in food selection 

Dear Dr. Lee:

I'm pleased to inform you that your manuscript has been deemed suitable for publication in PLOS ONE. Congratulations! Your manuscript is now with our production department. 

If your institution or institutions have a press office, please let them know about your upcoming paper now to help maximize its impact. If they'll be preparing press materials, please inform our press team within the next 48 hours. Your manuscript will remain under strict press embargo until 2 pm Eastern Time on the date of publication. For more information please contact onepress@plos.org.

If we can help with anything else, please email us at plosone@plos.org. 

Thank you for submitting your work to PLOS ONE and supporting open access. 

Kind regards, 

PLOS ONE Editorial Office Staff

on behalf of

Dr. John W. Apolzan 

Academic Editor

PLOS ONE
